# Louse-Borne Relapsing Fever (*Borrelia recurrentis*) in a Somali Refugee Arriving in Italy: A Re-emerging Infection in Europe?

**DOI:** 10.1371/journal.pntd.0004522

**Published:** 2016-05-05

**Authors:** Spinello Antinori, Oleg Mediannikov, Mario Corbellino, Romualdo Grande, Carlo Parravicini, Giovanna Bestetti, Erika Longhi, Davide Ricaboni, Cyrille Bilé Ehounoud, Florence Fenollar, Didier Raoult, Sara Giordana Rimoldi

**Affiliations:** 1 Department of Clinical and Biomedical Sciences Luigi Sacco, University of Milano, Milano, Italy; 2 III Division of Infectious Diseases, Luigi Sacco Hospital, Milano, Italy; 3 Unité de Recherche sur le Maladies Infectieuses et Tropicales Emergentes (URMITIE) IRD198, CNRS 7278, INSERM 1095, Institute Hospitalo-Universitaire (IHU) Mediterranee-Infection, Aix-Marseille Université, Faculté de Médecine, Marseille, France; 4 Department of Diagnostic Services, Clinical Microbiology, Virology and Bioemergence Diagnostics, Luigi Sacco Hospital, Milano, Italy; 5 Department of Diagnostic Services Pathology Unit, Luigi Sacco Hospital, Milano, Italy; Universidade do Estado do Rio de Janeiro, BRAZIL

## Introduction

Louse-borne relapsing fever (LBRF) is an acute febrile infection that is typically characterized by one to three fairly regular waves of bacteremia [1,2]. It is caused by *Borrelia recurrentis*, a motile spirochete that measures 5 to 40 μm in length. The microorganism is transmitted from person to person by the human body louse (*Pediculus humanus humanus*). Disruptions in sanitation during wartime and mass migrations of people provide conditions that favor the propagation of body lice and thus the occurrence of outbreaks of the disease [1,3]. LBRF is endemic in East Africa (e.g., Ethiopia, Eritrea, Somalia, and Sudan) with the highest number of cases observed in Ethiopia, where it is the seventh most common cause of hospital admission and the fifth most common cause of death [4,5]. We report here the first case of imported LBRF observed in Lombardy (northern Italy) in a Somali refugee.

## Case Presentation

**Fig 1:**
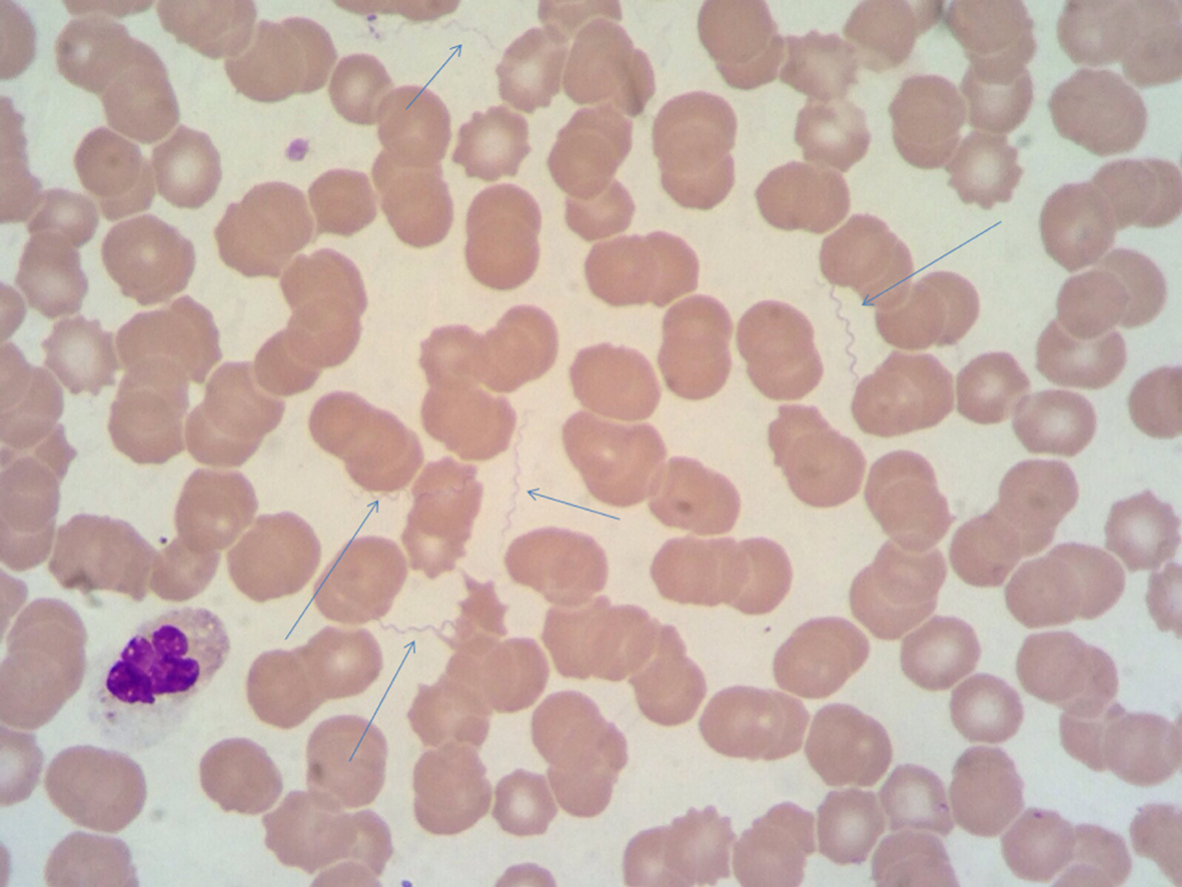
May-Grünwald-Giemsa (MGG) stained thin film showing numerous spirochetes (arrows) identified as *Borrelia recurrentis* (1,000-fold magnification).

On 13 September 2015, a 22-year-old refugee from Somalia presented to the Emergency Department (ED) of Luigi Sacco Hospital complaining of high fever, headache, chills, nausea, vomiting, and diffuse myalgias. He had arrived in Italy six days earlier after stopovers in Sudan (three weeks) and Libya (two months) on the way to our country. On the ninth of September he reported the onset of fever. On presentation, the patient was mildly febrile (37.3°C), tachypneic (respiratory rate 32 breaths per minute), hypotensive (90/60 mmHg), and showed bilateral hyperemic conjunctivae. The remainder of the physical examination was unremarkable. Routine blood tests demonstrated severe thrombocytopenia (23 x 10^9^/L), a normal leukocyte count with lymphocytopenia (7.3 x 10^9^/L; 6%), a C-reactive protein concentration of 440.9 mg/L (normal < 3 mg/L) and high venous lactate levels (3.9 mmol/L). Chest X-ray and abdominal ultrasound examination were reportedly normal. Thick and thin blood films were negative for malaria parasites, along with a rapid diagnostic antigen test. A second febrile peak (38.5°C) while the patient was in the ED and before inception of empirical antibiotic therapy with piperacillin/tazobactam (4.5 g ev) was remarkable due to the presence of a large number of extracellular spirochetes detected on blood film (Fig 1).

Serological screening tests for human immunodeficiency virus (HIV) and syphilis were negative. A western immunoblot for *Borrelia* species was positive for both IgG and IgM (p41, p39, OspC). The patient was admitted to our infectious diseases ward with a tentative diagnosis of borreliosis. No body lice were detected from his clothing, but it should be noted that they were changed upon arrival in Italy. He was also negative for the presence of head lice. Antibiotic therapy was switched to 100 mg of doxycycline twice per day and was stopped after three days. Jarisch-Herxheimer reaction did not occur. The patient made a full recovery, with platelet counts returning to normal values by the seventh day, and was discharged without consequences.

## Identification of Bacterial Agent

**Fig 2:**
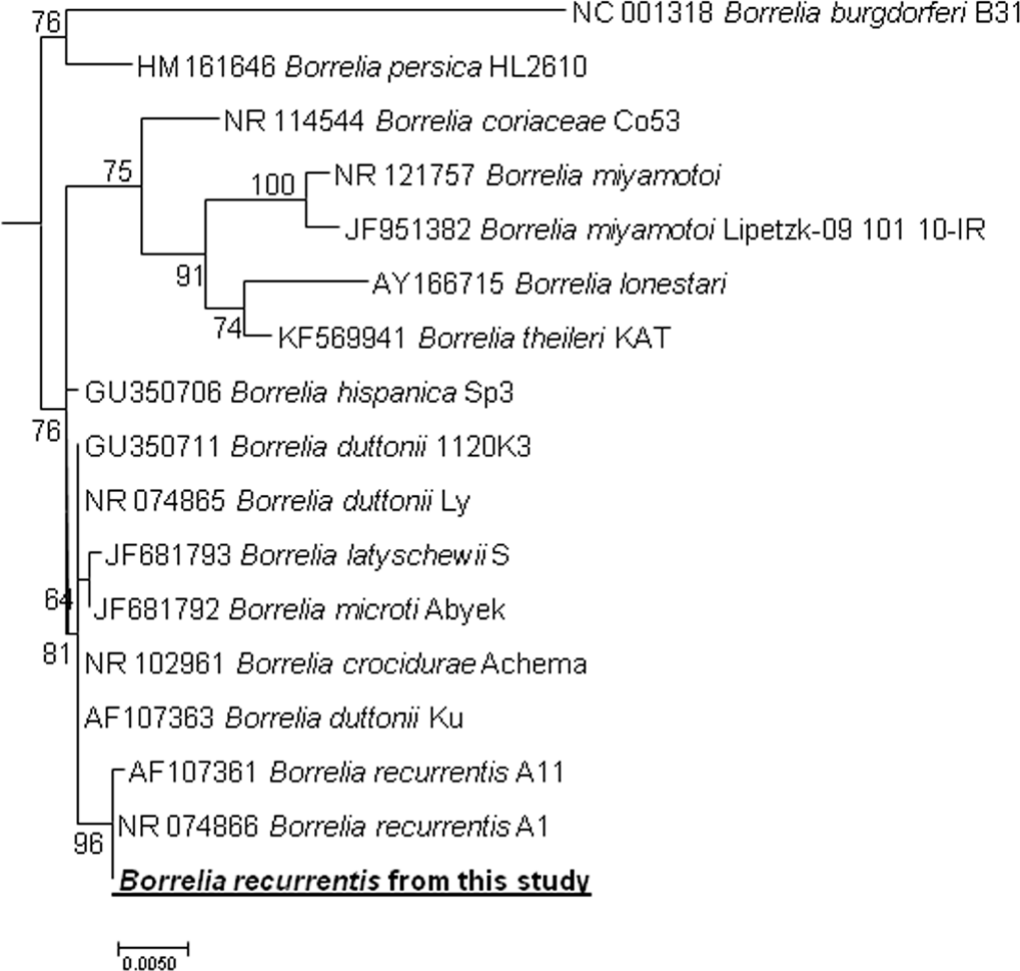
Phylogenetic tree highlighting the position of *Borrelia recurrentis* identified in the present study relative to *Borrelia* type strains and uncultured borreliae. The 16S rRNA gene sequences were aligned using CLUSTALW, and phylogenetic inferences were obtained from a maximum likelihood (ML) phylogenetic analysis with the TrN+Γ substitution model. The GenBank accession numbers are indicated at the beginning. Sequence obtained in the present study is in bold. The numbers at the nodes are the bootstrap values obtained by repeating the analysis 100 times to generate a majority consensus tree. There were a total of 1,269 positions in the final dataset. The scale bar indicates a 5% nucleotide sequence divergence.

Broad range 16S rRNA polymerase chain reaction (PCR) was performed on DNA extracted from the blood drawn before administration of antimicrobial therapy. A real-time PCR based on *rrs* (16S rRNA) gene and aimed to amplify all species of *Borrelia* [6] was also positive. In order to identify borrelial species causing the fever in this patient, we amplified a 344 bps portion of a *flaB* (flagellin) gene [7] and a 1395 bps portion of *rrs* using *Borrelia* genus-specific primers. Sequenced amplicons of both genes showed 100% identity (297/297 for *flaB* and 1269/1269 for *rrs*) with the *B*. *recurrentis* strain A1 CP000993 (Fig 2).

## Case Discussion

We report here the first case of imported LBRF diagnosed in northern Italy in a Somali refugee that was observed almost concomitantly with similar cases in East African refugees in the Netherlands, Switzerland [8,9], southern and northern Italy [10,11], and Germany [12] in a very limited time span.

Louse-borne relapsing fever is caused by the spirochete *B*. *recurrentis* with humans being the only reservoir [1]. It is usually transmitted by the rupture of a body louse with subsequent auto-inoculation caused by scratching of the skin due to presence of *Borrelia* in the hemolymph of the arthropod. The recent demonstration of prolonged excretion of the bacterium in the feces of the louse provides an alternative explanation for a more efficient means of transmission [2,13]. Although a study showed that *B*. *recurrentis* can be frequently identified in head lice (*Pediculus humanus capitis*) of patients with LBRF, the possible role of the latter in human transmission of the disease still remains to be demonstrated [14]. During World Wars I and II, epidemics of relapsing fever affected millions of people worldwide. Subsequently, the disease disappeared from western countries, while it still remains a fairly common and important disorder in Eastern Africa (especially in Ethiopia, Eritrea, Somalia, and Sudan) [4,5,15,16].

The ongoing “humanitarian crisis” with thousands of refugees and migrants daily arriving to the European Union after perilous journeys in crowded and poor hygienic conditions is a definite risk factor for possible explosive outbreaks of arthropod-borne diseases [3,17,18]. Language barriers, which hamper the acquisition of accurate clinical and travel history, coupled with unfamiliarity with these disorders are a challenge for western physicians. As in the case of this patient, all other individuals with LBRF identified in the European Union had travelled from the Horn of Africa through Sudan and Libya before arriving in Europe. These cases altogether suggest that *B*. *recurrentis* is currently circulating among East African refugees, most of whom first arrive in Italy as “boat people” after having waited for long periods of time in Libya. Since the incubation period of LBRF is relatively short (mean: 7 days, range: 2–18 days), we may hypothesize the existence of an “epidemic chain” that probably begins in Somalia and is then fueled by the “relapsing nature” of the microorganism, together with high rates of body louse infestation, crowding, and poor hygienic conditions that are well known to affect migrants and refugees in their long journeys to Europe. It is also likely that a new focal point of infection might have been established in Libya. The disease is characterized by signs and symptoms that are not specific and may mimic a sepsis-like syndrome or other febrile infectious diseases that are associated with thrombocytopenia (e.g., malaria, leptospirosis, rickettsiosis, meningococcal sepsis, or viral hemorrhagic fevers). Low platelet counts were indeed described in 11 of the 12 patients whose published cases were detailed [8–11], including the present one. The diagnosis of borreliosis is easily accomplished by the observation of spirochetes in the peripheral blood smear collected during febrile episodes due to the high bacterial load. However, we believe that these pathogens may go unnoticed to the inexperienced or unaware observer, or when blood samples are taken in the afebrile period or after starting antibiotic treatment. Finally, the identification of the microorganism at the species level should be actively pursued, because several tick-borne borreliae (e.g., *Borrelia duttonii*, *Borrelia hispanica*, and *Borrelia crocidurae*) have been documented in Africa [15,16]. Moreover, several species that are pathogenic for humans, but not yet completely characterized, were recently reported from Northern and Eastern Africa [19,20].

This is not a purely epidemiologic or microbiologic undertaking, but is of paramount therapeutic relevance because LBRF requires a single-dose antibiotic treatment, as opposed to a seven- to ten-day schedule for the tick-borne relapsing fevers. However, the correct identification of *Borrelia* at the species level is a task that can be accomplished only by reference laboratories using molecular tools and sequencing.

## Conclusion and Recommendation

Physicians should be aware of the possibility that initial therapy may precipitate a Jarisch-Herxheimer reaction, which was actually observed in 15 out of the 27 patients described up to now in Europe and probably caused one death [8–12]. Prompt recognition of LBRF among refugees from East Africa and appropriate antibiotic treatment are essential to avoid re-emergence of louse-borne infections in Europe and to reduce death from this disease, which can be as high as 20%–40% among malnourished and frail individuals. In all diagnosed cases of LBRF, clothes should be replaced or washed at high temperatures (>60°C). Management of body lice includes both bathing with soap and water and treatment with topical or systemic pediculicides. Close contacts of affected patients should be investigated for the presence of body lice and eventually deloused and/or treated with a single-dose prophylaxis of doxycycline.

**Key Learning Points**

- Louse-borne relapsing fever is increasingly observed among asylum seekers from the Horn of Africa arriving in Europe after prolonged stay in refugee camps in Libya.
- A sepsis-like picture associated with thrombocytopenia should raise the clinical suspicion of this disease.
- High bacterial load observed during febrile episodes makes the diagnosis straightforward when blood smears are examined, but the diagnosis may be missed by unskilled observers or when blood is collected after antibiotic treatment.
- A Jarisch-Herxheimer reaction might be precipitated by antibiotic treatment.
- Prompt recognition of this disease among refugees coming from East Africa and appropriate delousing are essential to avoid re-emergence of LBRF in Europe.
